# Supplementary material for: Decreased motor cortex excitability mirrors own hand disembodiment during the rubber hand illusion
Source: eLife. 2016 Oct 20;5:e14972. doi: 10.7554/eLife.14972 (PMC5072839; doi:10.7554/eLife.14972)
Supplement: Figure 2—source data 1. — (A) MAIN EXPERIMENT. For each subject, the proprioceptive drift (estimation of right index finger felt position) mean values, calculated as the difference between pre and post stimulation in synchronous (mean ± sd = 4.51 ± 4.2) and asynchronous (mean ± sd = 2.08 ± 2.75), are reported. (B) MAIN EXPERIMENT. For each subject, the mean rating value of the three ownership statements in synchronous (mean ± sd = 2.4 ± 0.64) and asynchronous (mean ± sd = -2.04 ± 0.9) are reported. DOI: http://dx.doi.org/10.7554/eLife.14972.005 [file elife-14972-fig2-data1.docx]

**Figure 2_source data 1**. Main experiment behavioral results following asynchronous and synchronous condition.

Section A.

| SUBJECT NUMBER | DRIFT | |
| --- | --- | --- |
|  | ASYNCHRONOUS | SYNCHRONOUS |
| 1 | 0,5 | -12,1 |
| 2 | -4,2 | -9 |
| 3 | -1,9 | -8,1 |
| 4 | -0,5 | -10,3 |
| 5 | -4,8 | -4,1 |
| 6 | -4,2 | -3,9 |
| 7 | 3,95 | -3,1 |
| 8 | -2,4 | -2,2 |
| 9 | -4,2 | -4,6 |
| 10 | -0,3 | -3,15 |
| 11 | -6,95 | -13,85 |
| 12 | -6,05 | -1,3 |
| 13 | -2,05 | -4,9 |
| 14 | -6,8 | -5,55 |
| 15 | -0,95 | -5,55 |
| 16 | -1,8 | 1,8 |
| 17 | -2,05 | -8,85 |
| 18 | 0,9 | -1,1 |
| 19 | 0,4 | -2,9 |
| 20 | 0,8 | 0,9 |
| 21 | 1,4 | 3,5 |
| 22 | -2,1 | -2,3 |
| 23 | -4,6 | -6,8 |
| 24 | -2,2 | -0,9 |

A). MAIN EXPERIMENT. For each subject, the proprioceptive drift (estimation of right index finger felt position) mean values, calculated as the difference between pre and post stimulation in synchronous (mean ± sd = 4.51 ± 4.2) and asynchronous (mean ± sd = 2.08 ± 2.75), are reported.

Section B.

| SUBJECT NUMBER | EMB-Q-RATING | |
| --- | --- | --- |
|  | ASYNCHRONOUS | SYNCHRONOUS |
| 1 | -3 | 3 |
| 2 | -1,6 | 2,7 |
| 3 | -2,3 | 3 |
| 4 | -2,7 | 3 |
| 5 | -0,3 | 2,7 |
| 6 | -2,7 | 1,7 |
| 7 | -2,3 | 2,7 |
| 8 | -1,7 | 2,3 |
| 9 | -1 | 3 |
| 10 | -1,6 | 2,3 |
| 11 | -0,6 | 1,6 |
| 12 | -2,6 | 1,3 |
| 13 | -0,3 | 2,7 |
| 14 | -2,8 | 2,7 |
| 15 | -3 | 2,5 |
| 16 | -3 | 1 |
| 17 | -2,3 | 1,3 |
| 18 | -3 | 3 |
| 19 | -3 | 3 |
| 20 | -1,7 | 3 |
| 21 | -1 | 1,7 |
| 22 | -1,3 | 2,3 |
| 23 | -2,3 | 3 |
| 24 | -3 | 2,3 |

B). MAIN EXPERIMENT. For each subject, the mean rating value of the three ownership statements in synchronous (mean ± sd = 2.4 ± 0.64) and asynchronous (mean ± sd = -2.04 ± 0.9) are reported.
