# Supplementary material for: Decreased motor cortex excitability mirrors own hand disembodiment during the rubber hand illusion
Source: eLife. 2016 Oct 20;5:e14972. doi: 10.7554/eLife.14972 (PMC5072839; doi:10.7554/eLife.14972)
Supplement: Figure 2—source data 2. — (A) CONTROL EXPERIMENT. For each subject, the proprioceptive drift (estimation of right index finger felt position) mean values, calculated as the difference between pre and post stimulation in synchronous (mean ± sd = 2.47 ± 2.707) and asynchronous (mean ± sd = 0.075 ± 2.461), are reported. (B) CONTROL EXPERIMENT. For each subject, the mean rating value of the three ownership statements in synchronous (mean ± sd = 2 ± 0.763) and asynchronous (mean ± sd = -0.97 ± 1.387) are reported. (C) CONTROL EXPERIMENT. For each subject, the mean rating value of the three disownership statements in synchronous (mean ± sd = 0.153 ± 1.427) and asynchronous (mean ± sd = -1.15 ± 1.371) are reported. DOI: http://dx.doi.org/10.7554/eLife.14972.006 [file elife-14972-fig2-data2.docx]

**Figure 2_source data 2**. Control experiment behavioral results following asynchronous and synchronous condition.

Section A.

| SUBJECT NUMBER | DRIFT | |
| --- | --- | --- |
|  | ASYNCHRONOUS | SYNCHRONOUS |
| 1 | 0,5 | -12,1 |
| 2 | -4,2 | -9 |
| 3 | -1,9 | -8,1 |
| 4 | -0,5 | -10,3 |
| 5 | -4,8 | -4,1 |
| 6 | -4,2 | -3,9 |
| 7 | 3,95 | -3,1 |
| 8 | -2,4 | -2,2 |
| 9 | -4,2 | -4,6 |
| 10 | -0,3 | -3,15 |
| 11 | -6,95 | -13,85 |
| 12 | -6,05 | -1,3 |
| 13 | -2,05 | -4,9 |
| 14 | -6,8 | -5,55 |
| 15 | -0,95 | -5,55 |
| 16 | -1,8 | 1,8 |
| 17 | -2,05 | -8,85 |
| 18 | 0,9 | -1,1 |
| 19 | 0,4 | -2,9 |
| 20 | 0,8 | 0,9 |

A). CONTROL EXPERIMENT. For each subject, the proprioceptive drift (estimation of right index finger felt position) mean values, calculated as the difference between pre and post stimulation in synchronous (mean ± sd = 2.47 ± 2.707) and asynchronous (mean ± sd = 0.075 ± 2.461), are reported.

Section B.

| SUBJECT NUMBER | EMB-Q-RATING | |
| --- | --- | --- |
|  | ASYNCHRONOUS | SYNCHRONOUS |
| 1 | -1,73333 | 2,333333 |
| 2 | -2,26667 | 2,6 |
| 3 | -1,2 | 0,733333 |
| 4 | 0,6 | 1,933333 |
| 5 | -1,8 | 1,466667 |
| 6 | 0,4 | 2,933333 |
| 7 | -2,86667 | 1,8 |
| 8 | 0,133333 | 1,333333 |
| 9 | 0,133333 | 2,933333 |
| 10 | -0,53333 | 2,333333 |
| 11 | -0,6 | 2,933333 |
| 12 | -2,93333 | 3 |
| 13 | -0,66667 | 2,533333 |
| 14 | -3 | 1,333333 |
| 15 | 0 | 1,4 |
| 16 | -1,2 | 0,733333 |
| 17 | -1,6 | 1,2 |
| 18 | 1,466667 | 3 |
| 19 | 1,066667 | 1,866667 |
| 20 | -2,8 | 1,6 |

B). CONTROL EXPERIMENT. For each subject, the mean rating value of the three ownership statements in synchronous (mean ± sd = 2 ± 0.763) and asynchronous (mean ± sd = -0.97 ± 1.387) are reported.

Section C.

| SUBJECT NUMBER | DISEMB-Q-RATING | |
| --- | --- | --- |
|  | ASYNCHRONOUS | SYNCHRONOUS |
| 1 | -2,8 | -0,33333 |
| 2 | -1,8 | 1,333333 |
| 3 | 0,8 | -1,33333 |
| 4 | 0,8 | 1,866667 |
| 5 | -2,2 | 0,933333 |
| 6 | -2,06667 | -1,66667 |
| 7 | -3 | -1,46667 |
| 8 | -0,33333 | -0,6 |
| 9 | -0,66667 | 0,2 |
| 10 | -0,53333 | -0,73333 |
| 11 | 1,2 | 2,466667 |
| 12 | -2,93333 | 2,066667 |
| 13 | -1,06667 | 0,266667 |
| 14 | -2 | -2,33333 |
| 15 | -1,46667 | 0,466667 |
| 16 | -0,86667 | 0,066667 |
| 17 | -1,86667 | -0,8 |
| 18 | 1 | 2,8 |
| 19 | -0,33333 | 0,533333 |
| 20 | -2,86667 | -0,66667 |

C). CONTROL EXPERIMENT. For each subject, the mean rating value of the three disownership statements in synchronous (mean ± sd = 0.153 ± 1.427) and asynchronous (mean ± sd = -1.15 ± 1.371) are reported.
