# Supplementary material for: Decreased motor cortex excitability mirrors own hand disembodiment during the rubber hand illusion
Source: eLife. 2016 Oct 20;5:e14972. doi: 10.7554/eLife.14972 (PMC5072839; doi:10.7554/eLife.14972)
Supplement: Figure 3—source data 1. — (A) MAIN EXPERIMENT. For each subject, the mean MEPs amplitude (row values in µV), recorded during baseline (mean ± sd = 945.204 ± 535.076), asynchronous condition (mean ± sd = 918.075 ± 635.777) and synchronous condition (mean ± sd = 527.328 ± 325.998), are reported. (B) MAIN EXPERIMENT. For each subject, the mean MEPs amplitude (normalized z-scores), recorded during baseline (mean ± sd = 0.277 ± 0.691), asynchronous condition (mean ± sd = 0.205 ± 0.395) and synchronous condition (mean ± sd = -0.367 ± 0.362), are reported. In the z-scores computation, the mean and the sd of the three conditions were used to normalized row data according to the formula x-mean/sd. (C) MAIN EXPERIMENT. For each subject, mean amplitude of 5 MEPs (normalized z-scores) at four time-point recorded during synchronous condition are reported (respectively mean ± sd of TIME 1, 2, 3, 4: -0.239 ± 0.425, -0.284 ± 0.620, -0.389 ± 0.385, -0.548 ± 0.394). DOI: http://dx.doi.org/10.7554/eLife.14972.008 [file elife-14972-fig3-data1.docx]

**Figure 3_source data 1**. Main experiment physiological results during baseline, asynchronous and synchronous condition.

Section A.

| SUBJECT NUMBER | MEPs ROW DATA | | |
| --- | --- | --- | --- |
|  | BASELINE | ASYNCHRONOUS | SYNCHRONOUS |
| 1 | 561 | 811,64 | 801,68 |
| 2 | 694,8 | 943,2917 | 1138,04 |
| 3 | 1850 | 1312,88 | 633,72 |
| 4 | 1783 | 3004,167 | 666,96 |
| 5 | 1510 | 482,44 | 439,28 |
| 6 | 503 | 1411,304 | 920,96 |
| 7 | 483 | 369,08 | 515,32 |
| 8 | 428 | 671,36 | 763,32 |
| 9 | 957 | 846,36 | 740 |
| 10 | 432,35 | 242,5 | 148,25 |
| 11 | 609,7 | 771,8 | 150,5 |
| 12 | 969,85 | 624,8 | 316,5 |
| 13 | 1215,7 | 2046,1 | 490,3 |
| 14 | 946,95 | 857,55 | 1152,15 |
| 15 | 1241,8 | 669,4 | 751,55 |
| 16 | 1489,95 | 678,15 | 295 |
| 17 | 207,2 | 370,45 | 252,7 |
| 18 | 661,25 | 371,6667 | 169,95 |
| 19 | 374,05 | 386,65 | 342,75 |
| 20 | 927,5 | 589,7 | 254,25 |
| 21 | 639,8 | 928,5 | 166,7 |
| 22 | 974 | 1101 | 270 |
| 23 | 2350 | 1882 | 1052 |
| 24 | 875 | 661 | 224 |

A). MAIN EXPERIMENT. For each subject, the mean MEPs amplitude (row values in µV), recorded during baseline (mean ± sd = 945.204 ± 535.076), asynchronous condition (mean ± sd = 918.075 ± 635.777) and synchronous condition (mean ± sd = 527.328 ± 325.998), are reported.

Section B.

| SUBJECT NUMBER | MEPs NORMALIZED (z-scores) | | |
| --- | --- | --- | --- |
|  | BASELINE | ASYNCHRONOUS | SYNCHRONOUS |
| 1 | -0,42411 | 0,051883 | 0,032938 |
| 2 | -0,56475 | -0,06979 | 0,182741 |
| 3 | 1,052666 | 0,343246 | -0,55378 |
| 4 | -0,0608 | 0,835054 | -0,82289 |
| 5 | 2,291407 | -0,17731 | -0,28097 |
| 6 | -0,96728 | 0,536247 | -0,34279 |
| 7 | 0,116954 | -0,24225 | 0,218864 |
| 8 | -0,65009 | -0,04871 | 0,178725 |
| 9 | 0,492022 | 0,133138 | -0,21186 |
| 10 | 0,857684 | -0,173 | -0,68468 |
| 11 | 0,228217 | 0,601767 | -0,82998 |
| 12 | 0,505347 | -0,0186 | -0,48675 |
| 13 | -0,03816 | 0,867136 | -0,82898 |
| 14 | -0,06002 | -0,19903 | 0,259052 |
| 15 | 0,521023 | -0,32093 | -0,20009 |
| 16 | 0,892735 | -0,19069 | -0,70204 |
| 17 | -0,57953 | 0,780109 | -0,20058 |
| 18 | 0,591923 | -0,15402 | -0,52967 |
| 19 | 0,034904 | 0,105459 | -0,14036 |
| 20 | 0,429712 | -0,001 | -0,42871 |
| 21 | 0,141291 | 0,804917 | -0,94621 |
| 22 | 0,260612 | 0,699118 | -0,70954 |
| 23 | 0,823774 | 0,378535 | -0,41149 |
| 24 | 0,775106 | 0,38331 | -0,41431 |

B). MAIN EXPERIMENT. For each subject, the mean MEPs amplitude (normalized z-scores), recorded during baseline (mean ± sd = 0.277 ± 0.691), asynchronous condition (mean ± sd = 0.205 ± 0.395) and synchronous condition (mean ± sd = -0.367 ± 0.362), are reported. In the z-scores computation, the mean and the sd of the three conditions were used to normalized row data according to the formula x-mean/sd.

Section C.

| SUBJECT NUMBER |  | SYNCHRONOUS MEP TIME COURSE PROFILE (z-scores) | | |
| --- | --- | --- | --- | --- |
|  | TIME 1 90s | TIME 2 180s | TIME 3 270s | TIME 4 360 s |
| 1 | -0,15223 | -0,10277 | 0,012306 | 0,325661 |
| 2 | 0,066375 | 0,781655 | -0,22053 | 0,114792 |
| 3 | -0,19195 | -0,46095 | -0,34715 | -1,12059 |
| 4 | -0,87852 | -0,7803 | -0,70701 | -0,91105 |
| 5 | 0,450441 | -0,75675 | -0,46936 | -0,33859 |
| 6 | -0,08001 | -0,48916 | -0,42317 | -0,37368 |
| 7 | 0,061248 | 1,113352 | 0,063875 | -0,27989 |
| 8 | 0,296647 | 0,292525 | 0,229871 | -0,06373 |
| 9 | -0,02589 | -0,17132 | -0,02102 | -0,5696 |
| 10 | -0,75987 | -0,89017 | -0,71427 | -0,37442 |
| 11 | -0,80763 | -0,6136 | -0,90811 | -0,9906 |
| 12 | 0,236502 | -0,7171 | -0,71588 | -0,7505 |
| 13 | -0,73511 | -0,55502 | -0,75234 | -1,27345 |
| 14 | 0,503254 | 0,626094 | 0,270325 | -0,36347 |
| 15 | 0,354515 | -0,52715 | -0,33476 | -0,29298 |
| 16 | -0,36412 | -0,88382 | -0,68736 | -0,87287 |
| 17 | -0,4546 | 0,25999 | -0,08481 | -0,5229 |
| 18 | -0,76561 | -0,53275 | -0,3072 | -0,51312 |
| 19 | -0,33047 | 0,866724 | -0,56005 | -0,53765 |
| 20 | -0,65548 | -0,2467 | -0,48207 | -0,3306 |
| 21 | -0,5644 | -1,15148 | -0,90736 | -1,16159 |
| 22 | -0,26955 | -0,83421 | -0,89957 | -0,81694 |
| 23 | -0,10159 | -0,58838 | -0,20774 | -0,70013 |
| 24 | -0,58296 | -0,45839 | -0,17575 | -0,43646 |

C). MAIN EXPERIMENT. For each subject, mean amplitude of 5 MEPs (normalized z-scores) at four time-point recorded during synchronous condition are reported (respectively mean ± sd of TIME 1, 2, 3, 4: -0.239 ± 0.425, -0.284 ± 0.620, -0.389 ± 0.385, -0.548 ± 0.394).
