# Supplementary material for: Decreased motor cortex excitability mirrors own hand disembodiment during the rubber hand illusion
Source: eLife. 2016 Oct 20;5:e14972. doi: 10.7554/eLife.14972 (PMC5072839; doi:10.7554/eLife.14972)
Supplement: Figure 3—source data 2. — (A) CONTROL EXPERIMENT. For each subject, the mean MEPs amplitude (row values in µV), recorded during baseline (mean ± sd = 725.665 ± 365.104), asynchronous condition (mean ± sd = 734.398 ± 416.262) and synchronous condition (mean ± sd = 685.368 ± 469.286), are reported. (B) CONTROL EXPERIMENT. For each subject, the mean MEPs amplitude (normalized z-scores), recorded during baseline (mean ± sd = 0.057 ± 0.368), asynchronous condition (mean ± sd = 0.045 ± 0.262) and synchronous condition (mean ± sd = -0.104 ± 0.399), are reported. In the z-scores computation, the mean and the sd of the three conditions were used to normalized row data according to the formula x-mean/sd. (C) CONTROL EXPERIMENT. For each subject, mean amplitude of 5 MEPs (normalized z-scores) at four time-point recorded during synchronous condition are reported (respectively mean ± sd of TIME 1, 2, 3, 4: −0.092 ± 0.483, −0.224 ± 0.435, −0.093 ± 0.656, −0.009 ± 0.587). DOI: http://dx.doi.org/10.7554/eLife.14972.009 [file elife-14972-fig3-data2.docx]

**Figure 3_source data 2**. Control experiment physiological results during baseline, asynchronous and synchronous condition.

Section A.

| SUBJECT NUMBER | MEPs ROW DATA | | |
| --- | --- | --- | --- |
|  | BASELINE | ASYNCHRONOUS | SYNCHRONOUS |
| 1 | 723,4 | 934,55 | 699,15 |
| 2 | 795,1 | 908,25 | 402,9 |
| 3 | 787,35 | 308,65 | 261,05 |
| 4 | 358,4 | 276,2 | 85,5 |
| 5 | 246,9 | 203,8 | 59,5 |
| 6 | 579,6 | 373,25 | 682,2 |
| 7 | 537,65 | 675 | 1130,1 |
| 8 | 1083,15 | 1428,35 | 1451,3 |
| 9 | 554,4 | 564,7 | 721,35 |
| 10 | 843,4 | 823,45 | 506,2 |
| 11 | 1377,35 | 1763 | 1987,25 |
| 12 | 490,45 | 678,35 | 366,1579 |
| 13 | 445,9 | 308,85 | 603,5 |
| 14 | 619,05 | 601,85 | 583,35 |
| 15 | 545,4 | 965,7 | 939,6 |
| 16 | 367 | 367,3 | 281,65 |
| 17 | 452,95 | 542,55 | 451,9 |
| 18 | 1159,15 | 838,9 | 735,05 |
| 19 | 861,45 | 757,95 | 579,15 |
| 20 | 1685,25 | 1367,3 | 1180,5 |

A). CONTROL EXPERIMENT. For each subject, the mean MEPs amplitude (row values in µV), recorded during baseline (mean ± sd = 725.665 ± 365.104), asynchronous condition (mean ± sd = 734.398 ± 416.262) and synchronous condition (mean ± sd = 685.368 ± 469.286), are reported.

Section B.

| SUBJECT NUMBER | MEPs NORMALIZED (z-scores) | | |
| --- | --- | --- | --- |
|  | BASELINE | ASYNCHRONOUS | SYNCHRONOUS |
| 1 | -0,20396 | 0,487312 | -0,28335 |
| 2 | 0,184021 | 0,407873 | -0,59189 |
| 3 | 0,920339 | -0,39478 | -0,52555 |
| 4 | 0,571348 | 0,174574 | -0,74592 |
| 5 | 0,265237 | 0,116451 | -0,38169 |
| 6 | 0,088743 | -0,44076 | 0,352021 |
| 7 | -0,50512 | -0,21993 | 0,725044 |
| 8 | -0,33084 | 0,149455 | 0,181387 |
| 9 | -0,11059 | -0,09131 | 0,201901 |
| 10 | 0,322921 | 0,268807 | -0,59173 |
| 11 | -0,48903 | 0,079282 | 0,409745 |
| 12 | -0,04657 | 0,32316 | -0,32717 |
| 13 | -0,01845 | -0,38764 | 0,406091 |
| 14 | 0,03447 | 0,000847 | -0,03532 |
| 15 | -0,49165 | 0,269456 | 0,222193 |
| 16 | 0,078775 | 0,079609 | -0,15838 |
| 17 | -0,09877 | 0,201051 | -0,10228 |
| 18 | 0,356812 | -0,10373 | -0,25308 |
| 19 | 0,224405 | 0,043799 | -0,2682 |
| 20 | 0,392062 | -0,0625 | -0,32956 |

B). CONTROL EXPERIMENT. For each subject, the mean MEPs amplitude (normalized z-scores), recorded during baseline (mean ± sd = 0.057 ± 0.368), asynchronous condition (mean ± sd = 0.045 ± 0.262) and synchronous condition (mean ± sd = -0.104 ± 0.399), are reported. In the z-scores computation, the mean and the sd of the three conditions were used to normalized row data according to the formula x-mean/sd.

Section C.

| SUBJECT NUMBER |  | SYNCHRONOUS MEP TIME COURSE PROFILE (z-scores) | | |
| --- | --- | --- | --- | --- |
|  | TIME 1 90s | TIME 2 180s | TIME 3 270s | TIME 4 360 s |
| 1 | 0,128008 | -0,85022 | -0,37355 | -0,03765 |
| 2 | -0,55252 | -0,84611 | -0,7923 | -0,17664 |
| 3 | -0,32569 | -0,6147 | -0,13228 | -1,02954 |
| 4 | -0,84101 | -0,48865 | -0,953 | -0,70103 |
| 5 | -0,10448 | -0,45038 | -0,4856 | -0,48629 |
| 6 | -0,13964 | 0,00047 | 0,645579 | 0,901673 |
| 7 | 0,365204 | 0,72276 | 0,779238 | 1,032975 |
| 8 | -0,25703 | -0,2676 | 1,330508 | -0,08033 |
| 9 | 1,070492 | -0,27044 | 0,253281 | -0,24573 |
| 10 | -0,72898 | -0,64272 | -0,38612 | -0,60909 |
| 11 | 0,576487 | 0,564698 | 0,190099 | 0,307696 |
| 12 | 0,464147 | -0,73498 | -0,81487 | -0,22298 |
| 13 | 0,458082 | -0,02626 | 0,386426 | 0,806121 |
| 14 | -0,18496 | -0,07432 | -0,15876 | 0,27677 |
| 15 | -0,47897 | 0,134547 | 0,896196 | 0,337002 |
| 16 | -0,19965 | 0,182698 | -0,14685 | -0,46973 |
| 17 | -0,33217 | -0,10262 | -0,91039 | 0,936045 |
| 18 | -0,23791 | -0,47231 | 0,102919 | -0,40501 |
| 19 | 0,062383 | 0,08786 | -0,81779 | -0,40527 |
| 20 | -0,58447 | -0,33743 | -0,47782 | 0,081467 |

C). CONTROL EXPERIMENT. For each subject, mean amplitude of 5 MEPs (normalized z-scores) at four time-point recorded during synchronous condition are reported (respectively mean ± sd of TIME 1, 2, 3, 4: -0.092 ± 0.483, -0.224 ± 0.435, -0.093 ± 0.656, -0.009 ± 0.587).
