# Supplementary material for: Decreased motor cortex excitability mirrors own hand disembodiment during the rubber hand illusion
Source: eLife. 2016 Oct 20;5:e14972. doi: 10.7554/eLife.14972 (PMC5072839; doi:10.7554/eLife.14972)
Supplement: Supplementary file 1. — DOI: http://dx.doi.org/10.7554/eLife.14972.010 [file elife-14972-supp1.docx]

**Power analysis**

When we planned the study, the sample size was evaluated according to previous literature on both behavioral effect of the RHI and MEPs modulation during cognitive task. With respect to the RHI, we based our sample size estimation on previous data acquire in our lab and then published in Burin et al., 2015. In that study, we replicate the RHI effect (using both the proprioceptive drift and the body-ownership questionnaire as objective and subjective measures of the illusion) in a group of seventeen (three subjects form the original samples of twenty were excluded as outliers) right-handed healthy subjects, tested as control group for brain-damage patients. With respect to the MEPs modulation, we selected a well-replicated task of Avenanti and colleagues, investigating changes in the corticospinal excitability during the observation of a hand-model receiving pain (Avenanti et al., 2005; 2006; 2009; 2010). As indicated in Avenanti et al., 2009, the effect of condition in modulating the MEP amplitude has a Cohens’s d = 0.81 (modified for within-subject design). G-power software (Heinrich Heine University - Institut für experimentelle psychologie; www.psycho.uni-duesseldorf.de/abteilungen/aap/gpower3) was used to estimate, in an a-priori analysis, the sample size in a paired T test (two tail), using the Avenanti’s Cohens’ d = 0.81; α = 0.05; Power (1-β err prob) = 0.95. A total sample size of 22 subjects was indicated. Thus, based on both our previous RHI paper (20 subjects) and this power analysis result (22 subjects), we estimated a sample size conservatively above these numbers and we recruited 26 subjects for the study.

**References for power analysis**

Avenanti A, Bueti D, Galati G, Aglioti SM (2005) Transcranial magnetic stimulation highlights the sensorimotor side of empathy for pain. Nature Neuroscience doi:10.1038

Avenanti A, Minio-Paluello M, Bufalari I, Aglioti SM (2006) Stimulus-driven modulation of motor-evoked potentials during observation of others’ pain. NeuroImage 32, 316 – 324, doi: 10.1016/j.neuroimage.2006.03.010

Avenanti A, Minio-Paluello M, Bufalari I, Aglioti SM (2009) The pain of a model in the personality of an onlooker: Influence of state-reactivity and personality traits on embodied empathy for pain. NeuroImage 44, 275, doi:10.1016/j.neuroimage.2008.08.001

Avenanti A, Sirigu A, Aglioti A (2010) Report racial bias reduces empathic sensorimotor resonance with other-race pain. Current Biology 20, 1018–1022, doi 10.1016/j.cub.2010.03.

Burin D, Livelli A, Garbarini F, Fossataro C, Folegatti A, Gindri P, Pia L (2015) Are movements necessary for the sense of body ownership? Evidence from the rubber hand illusion in pure hemiplegic patients. *PLoS ONE* 10(3): e0117155HH.
